# Biomedicalização e as respostas à aids no Brasil: notas de pesquisa

**DOI:** 10.1590/S0104-59702024000100049

**Published:** 2024-10-11

**Authors:** Simone Monteiro, Mauro Brigeiro

**Affiliations:** i Pesquisadora em saúde pública, Laboratório de Educação em Ambiente e Saúde/Instituto Oswaldo Cruz/Fiocruz. Rio de Janeiro – RJ – Brasil msimone@ioc.fiocruz.br; ii Pesquisador bolsista, Laboratório de Educação em Ambiente e Saúde/Instituto Oswaldo Cruz/Fiocruz. Rio de Janeiro – RJ – Brasil maurobrigeiro@hotmail.com

**Keywords:** Medicalização, Prevenção, Aids, Ciências Sociais, Brasil, Medicalization, Prevention, Aids, Social Sciences, Brazil

## Abstract

O texto aborda o processo de biomedicalização das respostas ao vírus da imunodeficiência humana (HIV) e à síndrome da imunodeficiência adquirida (aids), tendo por base pesquisas de campo no Rio de Janeiro e revisões documentais e bibliográficas. Informados pela perspectiva socioantropológica e pelos estudos sociais da ciência, argumentamos que o atual predomínio de estratégias biomédicas preventivas implica reconfigurações de ordens científica, institucional e das intervenções em saúde e da sexualidade. Tais transformações apontam para: a consolidação da definição da epidemia como um problema clínico, uma reconfiguração dos debates sobre sexualidade e estigma e um apagamento da discussão das desigualdades estruturais e dos direitos humanos. O processo de biomedicalização exige novas investigações sobre HIV/aids no campo das ciências sociais e humanas.

## Abstract (en)

This text addresses the process of biomedicalization in the responses to the human immunodeficiency virus (HIV) and acquired immunodeficiency syndrome (aids), based on field research in Rio de Janeiro and document and literature reviews. Considering the socio-anthropological perspective and social studies of science, the current predominance of preventative biomedical strategies implies both reconfigurations of the scientific and institutional order and interventions in health and sexuality. These transformations reveal an established definition of the epidemic as a clinical problem, reconfiguration of debates on sexuality and stigma, and erasure of the discussion on structural inequalities and human rights, and new studies on HIV/aids within social sciences and humanities are needed.

Uma das principais características da resposta global ao vírus da imunodeficiência humana (HIV) na última década é o predomínio de estratégias biomédicas, em detrimento de abordagens estruturais e comportamentais. Sob a perspectiva histórica, a reconfiguração é notória. A epidemia de HIV tornou-se um problema prioritariamente médico, a clínica vem a ser o lócus privilegiado para seu controle, e os debates acerca das desigualdades estruturais e dos direitos humanos perderam centralidade. A prevenção, agora englobada pela lógica do tratamento, supõe um esforço para rastreamento de casos, rápida vinculação a terapias antirretrovirais e oferta de profilaxias medicamentosas para pessoas consideradas sob risco. Este texto aborda desdobramentos dessas diretrizes globais no Brasil, articulando dados de nossas pesquisas recentes, informadas pela análise socioantropológica e pelos estudos sociais da ciência e da tecnologia.

O contraponto histórico com a atualidade pode ser encontrado na produção acadêmica sobre a construção social da síndrome da imunodeficiência adquirida (aids). Após o seu surgimento nos anos 1980, as respostas à epidemia se caracterizaram por relacionar políticas públicas e ativismo social, politizando a sexualidade e evocando a linguagem dos direitos humanos. Debates sobre acesso universal à saúde, poder médico, discriminação de gênero e estigma sexual, entre outros, informavam a produção de conhecimento e a formulação de políticas públicas. O advento da aids produziu uma configuração transnacional única na história contemporânea, marcada por tensões, interlocução e cooperação entre diversas instituições e atores sociais, tendo como fundamento a consciência das dimensões políticas e sociais da epidemia (Bastos, 2002; Cueto, Lopes, 2023).

Na última década, a partir de evidências clínicas, medicamentos que outrora transformaram a infecção pelo HIV em evento crônico e controlável passaram a ser usados para fins de prevenção, como ilustram as profilaxias pré- e pós-exposição ao HIV.^1^ A testagem do HIV, associada ao aconselhamento e definida pelo princípio da excepcionalidade, é hoje regular e rotinizada em conformidade com a estratégia do Tratamento como Prevenção (TcP), qual seja: ampliar acesso aos testes rápidos de HIV, dentro e fora dos serviços de saúde (praças, unidades móveis, boates, domicílio), visando identificar precocemente os casos positivos, encaminhá-los ao tratamento e, consequentemente, reduzir a circulação do vírus na população e alcançar o suposto fim da aids (Parker, 2023).

A introdução dos testes rápidos e das profilaxias é crucial para entender o processo de biomedicalização em curso. No conjunto de verdades científicas, a centralidade dessas tecnologias é indissociável de sua afirmação nas políticas globais de controle do HIV, formuladas e atualizadas pelo Programa das Nações Unidas sobre HIV/aids (Unaids, 2014, 2021), visando eliminar a aids até 2030. Argumentamos que as atuais diretrizes envolvem uma virada biomédica, representativa do processo contemporâneo de biomedicalização. Segundo Clarke et al. (2003), a medicalização revela a expansão da medicina e de sua racionalidade sobre domínios da vida social, reconstruindo-os e redefinindo-os. A biomedicalização vai além, indicando as transformações que os recursos tecnocientíficos operam sobre fenômenos médicos como doenças, disfunções, saúde e capacidades corporais, brindando-lhes novos sentidos. Essa nova configuração diz respeito a processos de medicalização mais complexos, multissituados e difusos, em contextos sociais intensamente condicionados pela produção científica e tecnológica, operando reordenamentos dos conhecimentos, instituições e práticas em saúde e da posição social de seus agentes. A saúde se torna uma obrigação moral, o corpo sendo o alvo de intervenções de autogoverno em nome do bem-estar e a matéria-prima da produção de identidades derivadas de verdades biotécnicas. Integram esse processo a cooptação de formas de conhecimentos concorrentes à biomedicina e a criação de novos procedimentos de legitimação das verdades.

As transformações no domínio da política global de controle da aids e a remodelação das respostas biomédicas à epidemia, nas duas últimas décadas, têm atraído significativo interesse acadêmico. Embora adotando conceitos particulares (farmaceuticalização, remedicalização e biomedicalização), as análises sublinham que a centralidade dos recursos biomédicos de prevenção do HIV e das respostas farmacológicas aos problemas de saúde pública têm como efeito a reorganização das práticas institucionais, dos serviços de saúde e do autocuidado, reformulando as concepções de saúde, doença e prevenção (Biehl, 2007; Nguyen et al., 2011; Clarke et al., 2003; Kenworthy, Thomann, Parker, 2017; Kippax, Stephenson, 2016; Monteiro et al., 2019).

Se, por um lado, as respostas à epidemia derivadas do avanço tecnológico biomédico representam tendências globais, por outro, assumem um caráter diferenciado ao situar-se em contextos específicos. Considerando esse panorama teórico e o alinhamento do governo brasileiro às políticas internacionais de aids, temos analisado os desenvolvimentos tecnocientíficos que hoje definem a resposta à epidemia no país.

## Implicações e efeitos da virada biomédica no âmbito local

As atuais diretrizes nacionais preconizam a combinação de intervenções biomédicas, comportamentais e estruturais, presentes na concepção de prevenção combinada (Brasil, 2017). Todavia, por meio de estudos socioantropológicos, desenvolvidos entre 2017 e 2021, constatamos que o aumento da oferta do teste rápido de HIV, profilaxia pós-exposição (PEP) e profilaxia pré-exposição (PrEP) tem predominado nas ações de prevenção no âmbito programático. Verificamos que o avanço dessas novas estratégias biomédicas ocorre a expensas da redução de intervenções históricas como: estímulo ao uso de preservativo, aconselhamento vinculado à testagem, estratégias de redução de danos, campanhas educativas regulares e enfrentamento das condições de vulnerabilidade ao HIV e do estigma da diversidade sexual e da aids. Tais estudos resultaram de revisões bibliográficas, análise documental, observação participante nos serviços de saúde de cinco municípios da zona metropolitana do Rio de Janeiro, bem como de entrevistas com a equipe de profissionais dos serviços, gestores estaduais e municipais, ativistas de ONG-aids e LGBT e *gays*/homens que fazem sexo com homens (HSH), mulheres trans, travestis e trabalhadores(as) sexuais usuários(as) de PEP e/ou PrEP (Monteiro, Brigeiro, 2018).^2^

De acordo com os achados, a distribuição de preservativos vem ocorrendo apenas em datas específicas, e são escassas as campanhas e intervenções educativas comunitárias, mesmo entre grupos considerados mais vulneráveis ao HIV (HSH, mulheres trans e trabalhadores/as sexuais). Segundo relatos de alguns gestores, as ações vêm progressivamente assumindo um tom mais biomédico e burocrático, acompanhadas pela falta de visibilidade da epidemia na agenda pública. Parcerias com organizações não governamentais (ONGs) se mostram esporádicas e centradas no recrutamento de pessoas para testagem e PrEP, havendo dificuldade de sustentabilidade do movimento social, decorrente da redução de financiamentos^3^ (Monteiro, Brigeiro, 2019). Temos ciência de que os recursos biotecnológicos ampliam a qualidade e a manutenção da vida das pessoas e contribuem para o controle da epidemia. Entretanto, os dados apontam que os investimentos nessas estratégias não têm sido acompanhados de ações preventivas comportamentais e estruturais, historicamente relevantes. Essas reconfigurações programáticas ilustram os efeitos do processo de biomedicalização.

Tal fenômeno tem mobilizado a análise social da epidemia no novo milênio. Um dos principais argumentos defendidos por diferentes autores é o da suposta oposição entre dimensões biomédicas e sociais. Afim aos estudos sociais da ciência e da tecnologia, Kippax e Stephenson (2016) e Race (2018) demonstram a artificialidade dessa diferenciação, uma vez que todas as intervenções de prevenção estão necessariamente envolvidas no cotidiano das pessoas e integradas às suas relações, sendo indissociáveis das práticas sociais. Portanto, críticas sobre as limitações de abordagens educativas/comunitárias ou as promessas das respostas biomédicas para contornar problemáticas sociais complexas seriam sempre enviesadas.

Nessa direção, cabe citar nosso trabalho sobre a trajetória social da PrEP no país, incluindo os processos de construção pública e a sua oferta no Sistema Único de Saúde (SUS). O estudo foi desenvolvido a partir de trabalho de campo, entrevistas com gestores, profissionais e ativistas no estado do Rio de Janeiro e da análise de diretrizes governamentais, protocolos clínicos, boletins de ONGs e textos da mídia. Segundo os achados, as narrativas relativas à PrEP sugerem tanto a esperança, associada ao controle da epidemia, à liberdade e ao prazer sexual, quanto receios e dúvidas sobre as suas implicações nas práticas cotidianas e na gestão da sexualidade. Tal análise permitiu identificar como as emoções de esperança e medo informam os sentidos atribuídos à PrEP e as negociações para implementação no âmbito programático (Brigeiro, Monteiro, 2022).

Com base nas observações nos serviços e entrevistas com usuários(as) de PrEP e PEP, identificamos o predomínio de homens *gays*, com altas e médias renda e escolaridade, havendo uma demanda reduzida de *gays*/HSH de baixo poder aquisitivo, mulheres trans, travestis e trabalhadoras sexuais (Silva Jr., Brigeiro, Monteiro, 2022, 2023; Murray, Brigeiro, Monteiro, 2022). Esses dados confirmam um problema apontado por gestores e profissionais consultados e atestado pela literatura nacional e internacional. Sugerem ainda que o apelo à prevenção biomédica pode ser mais ou menos efetivo conforme a posição de classe e as experiências de discriminação e estigma dos sujeitos. As diferenças de adesão às profilaxias de prevenção refletem igualmente a escassez de campanhas e intervenções comunitárias (Mora, Nelvo, Monteiro, 2022). Segundo os relatos de profissionais e usuários(as), em contraste com a PrEP, a PEP tende a ser significada como a remediação de um descuido, embora tecnicamente seja definida como intervenção emergencial e menos como um recurso preventivo.

O crescente processo de biomedicalização pode ser identificado ainda no modo como conceitos das ciências sociais têm sido cooptados na produção acadêmica sobre HIV, como ilustra nossa recente revisão bibliográfica acerca da testagem rápida entre HSH, mulheres trans/travestis e trabalhadoras sexuais.^4^ Nos artigos sobre barreiras de acesso ao teste entre HSH, os principais obstáculos descritos decorrem dos receios das repercussões do resultado positivo, de ser visto em locais de testagem, da associação da aids com práticas sexuais estigmatizadas e da dificuldade de compartilhar sua sexualidade com profissionais de saúde. Ao constatar que o estigma sexual dificulta o acesso de *gays*/HSH aos serviços de prevenção e testagem, os estudos, em geral, apontam para a necessidade de criar condições de acolhimento e garantia do anonimato e privacidade. Isso inclui, entre outras estratégias, distribuição de autoteste, envio domiciliar de *kit* teste, vinculação com serviços, campanhas na mídia social, envolvimento de organizações comunitárias no estímulo ao teste. O conceito de estigma é reduzido a uma barreira de acesso ao teste, em vez de suscitar debates sociais mais amplos sobre os efeitos negativos da discriminação sexual na prevenção do HIV. Igualmente, a noção de direitos tende a se resumir ao direito de acesso à testagem, perdendo seu potencial político.

É compreensível que o sigilo envolvendo o teste colabore para minimizar as experiências de sofrimento associadas à discriminação, como atestado pela literatura. Todavia, esse enfoque não privilegia reflexões sobre a função social do estigma na (re)produção das desigualdades sociais e da vulnerabilidade ao HIV (Parker, 2013). As estratégias recomendadas para avançar na superação do estigma apontam menos para uma transformação dos valores e moralidades sexuais e mais para a garantia do sigilo no acesso ao teste, buscando evitar a visibilidade indesejada.

## Considerações finais

A biomedicalização é um fenômeno crucial da história da aids. Com o predomínio da perspectiva clínica na prevenção, há uma expansão da autoridade e da prática médica, assim como do poder da indústria farmacêutica. Ao priorizar o âmbito do tratamento e dos espaços clínicos, ocorre a perda do protagonismo dos ativistas e das populações afetadas na definição e participação nas políticas de enfrentamento do HIV/aids, reduzindo as dimensões políticas, socioculturais e econômicas das respostas à epidemia (Kippax, Stephenson, 2016; Monteiro et al., 2019; Cueto, Lopes, 2023). Noções como prevenção são ressignificadas, sendo inclusive englobadas pela noção de tratamento.

Ademais, tal processo vem ocorrendo concomitantemente ao apagamento da aids na agenda pública, ao recrudescimento do discurso conservador e ao desmonte das políticas de saúde sexual e reprodutiva e direitos humanos do governo federal de 2019 a 2022, sendo acentuado pela recente pandemia de covid-19. As consequências são diversas e podem ser ilustradas por pesquisas atuais com adolescentes e jovens de baixa renda de cidades brasileiras, que atestam falta de conhecimento sobre sexualidade, contracepção e prevenção do HIV e demais infecções sexualmente transmissíveis (IST), ausência de fontes confiáveis de informação e de espaços para diálogo e aprendizagem sobre essas temáticas, e a maior preocupação com a gravidez do que com a HIV (Brasil, 2023).

Em convergência com nossos apontamentos, Brandt (2013) argumenta que a aids trouxe inovações nos conhecimentos convencionais no campo da saúde pública, nas práticas de investigação, nas atitudes culturais e nos comportamentos, inventando a saúde global. Com a biomedicalização, verificamos a emergência de novas reconfigurações no campo que comprometem as respostas sociais e estruturais à aids. Diante da atual conformação política e socioeconômica, nacional e global, fica o convite para que pesquisas nas áreas da história, sociologia, antropologia e saúde coletiva explorem o modo como futuros avanços no campo biomédico seguirão definindo as respostas à epidemia, as noções relativas ao processo saúde-doença, a subjetividade e as práticas sociais.

## NOTAS

1 A profilaxia pré-exposição (PrEP) envolve o uso regular de antirretrovirais antes da exposição ao HIV. No protocolo do Ministério da Saúde de 2017, é indicada para populações com maior prevalência de HIV (HSH, trans, trabalhador/a sexual) e casais sorodiscordantes. Na versão revisada do protocolo, a indicação da PrEP foi ampliada para “todos os adultos e adolescentes sexualmente ativos sob risco aumentado de infecção pelo HIV” (Brasil, 2022). A profilaxia pós-exposição (PEP), prevista nos casos de acidente profissional, violência sexual e sexo desprotegido, deve ser usada até 72 horas após a exposição ao HIV (Brasil, 2021). Ambas são oferecidas pelo SUS; a PrEP, desde dezembro de 2017, e a PEP, há mais de 12 anos.

2 A publicação apresenta os objetivos, a metodologia e os resultados do estudo qualitativo do Projeto Teste Perto de Você, fruto da parceria da Fiocruz (Escola Nacional de Saúde Pública e Instituto Oswaldo Cruz) com a Secretaria Estadual de Saúde-RJ, a Aids Health Care Foundation e os programas municipais de DST/IST/aids de Duque de Caxias, São João de Meriti, Belford Roxo, Nova Iguaçu e Niterói. O livro visa facilitar a apropriação dos achados da pesquisa por parte dos gestores, profissionais dos serviços e lideranças comunitárias, contribuindo para o processo de tomada de decisão.

3 Em anos recentes, os editais governamentais e internacionais para ONG-aids priorizaram o recrutamento das populações prioritárias para teste e PrEP, comprometendo seu histórico papel na formulação de políticas e controle social.

4 Trata-se de uma revisão integrativa, envolvendo 428 artigos publicados nas bases PubMed, SciELO, Sociological-Abstract, Assia, Web of Science e Scopus, de 2010 a 2019. Os trabalhos foram classificados segundo diversas categorias temáticas: barreiras de acesso ao teste, estratégias de captação, promoção da saúde, tratamento como prevenção, prevenção no marco dos Direitos Humanos, entre outras. Encontramos predomínio de artigos sobre *gays*/HSH em comparação com as demais populações e de estudos sobre barreiras de acesso ao teste, evocando a discussão do estigma (Brasil, 2023).
